# Ductus Venosus Agenesis: A Case Report and Review of the Literature

**DOI:** 10.7759/cureus.106678

**Published:** 2026-04-08

**Authors:** Marie Deckx, Amélie Boute, Emmanuel Bollue, Caroline Delforge

**Affiliations:** 1 Obstetrics and Gynaecology, Centre Hospitalier Universitaire Université Catholique de Louvain (CHU UCL) Namur - Site De Sainte-Elisabeth, Namur, BEL

**Keywords:** ductus venosus agenesis, fetal outcome, perinatal management, prenatal ultrasound diagnosis, vascular abnormalities

## Abstract

Ductus venosus agenesis (DVA) is a rare congenital anomaly of the fetal circulation defined by an absence of the physiological shunt connecting the hepatic-systemic system with the portal-umbilical venous system. Diagnosis relies on ultrasound and color Doppler imaging, mainly during the second trimester. We report the case of a fetus in a 34-year-old patient in whom DVA was diagnosed at 30 weeks gestation. Ultrasound revealed an extrahepatic umbilical vein draining directly into the right atrium that was associated with cardiomegaly. Molecular karyotyping was normal. Weekly ultrasound examinations demonstrated stability of the cardiomegaly during the remainder of the pregnancy. Neonatal outcomes were favorable, with good postnatal adaptation. DVA is a rare anomaly with variable prognosis determined mainly by the venous drainage subtype, the presence of associated malformations or genetic abnormalities, and the occurrence of intrauterine complications. Associated malformations, particularly cardiac anomalies, may be present. Given the possible association of DVA with genetic abnormalities, prenatal genetic testing may be considered. Potential complications such as cardiomegaly, fetal hydrops, or cardiac dysfunction highlight the importance of close echographic surveillance. Perinatal management of DVA requires an individualized approach that integrates clinical, sonographic, and genetic findings, with close ultrasound monitoring and planned delivery in a specialized center to optimize obstetric management, parental counseling, and neonatal outcomes.

## Introduction

Ductus venosus agenesis (DVA) describes a relatively rare congenital anomaly resulting from a failure of the connection between the hepatic-systemic and portal-umbilical venous systems [1]. DVA encompasses malformations characterized by the absence or abnormal course of the ductus venosus [2]. The clinical relevance of DVA derives from its heterogeneous presentation, its highly variable prognosis, and its potential association with structural and genetic abnormalities, which may significantly impact prenatal counseling and perinatal management.

Unlike the adult circulation system, the fetal circulation system contains three physiological shunts essential for intrauterine life: the ductus venosus, ductus arteriosus, and foramen ovale. These shunts play an essential role, creating preferential circulations that allow the fetus to adapt to relative hypoxemia and optimize placental exchange [3,4]. The ductus venosus bypasses the hepatic circulation, channeling a significant portion of oxygenated umbilical venous blood to flow directly into the inferior vena cava. This oxygen-rich blood is then preferentially directed to the left heart through the foramen ovale, supplying highly oxygenated blood to vital organs, such as the brain and heart [4,5].

During the first trimester, ultrasound and Doppler evaluation of the ductus venosus may be included in combined screening for trisomy 21 between 11 and 13 weeks of gestation. This screening combines nuchal translucency measurement, maternal serum biomarkers (beta-human chorionic gonadotropin (ß-hCG) and pregnancy-associated plasma protein A (PAPP-A)), and maternal age [6]. According to the Fetal Medicine Foundation, venous duct analysis should not be performed systematically but is useful in patients at intermediate risk (between 1/51 and 1/1000) as it can increase the sensitivity of the combined screening test [6]. Abnormal flow in the ductus venosus is associated with an increased risk of chromosomal anomalies, including trisomy 21, and congenital heart defects [1]. However, current literature provides limited support for the routine systematic screening of the ductus venosus during the first trimester.

During the second trimester, evaluation of the ductus venosus is particularly relevant in fetuses with intrauterine growth restriction [7]. Nevertheless, the increasing routine use of color Doppler during mid-trimester anomaly scans has led to more frequent detection of ductus venosus anomalies [1,2].

Here, we report a case of DVA diagnosed prenatally by ultrasound and review the literature to summarize its obstetric and prenatal features.

## Case presentation

The fetus of a 34-year-old multiparous patient was diagnosed with DVA at 30 weeks of gestation. The first-trimester ultrasound had been strictly normal with a nuchal translucency of 0.8 mm. The routine second-trimester ultrasound raised suspicion of an atrial septal defect.

**Figure 1:**
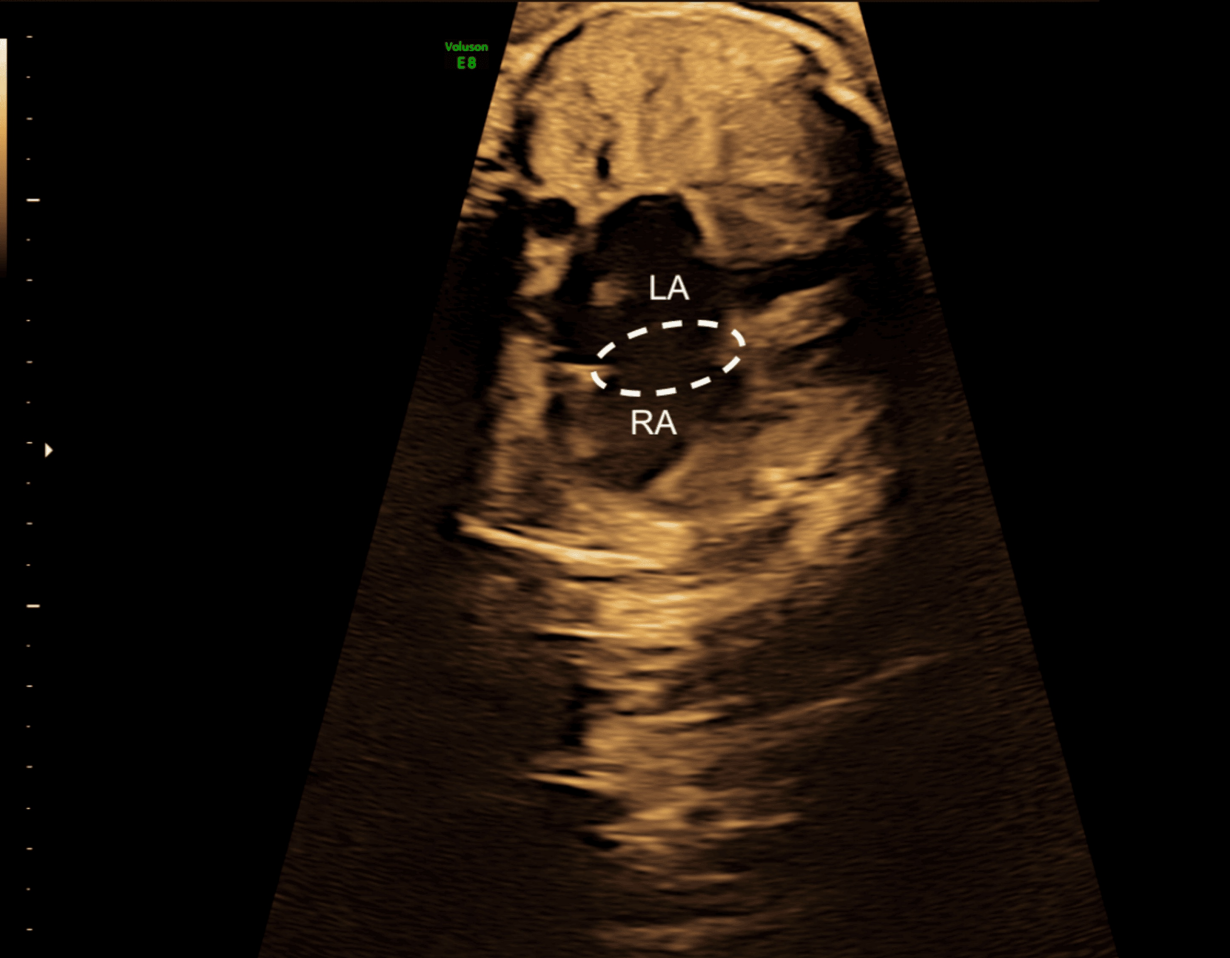
Ultrasound at 30 weeks + 0 days demonstrating a shunt between the right atrium (RA) and left atrium (LA) through a large foramen ovale (dashed outline), raising suspicion for an atrial septal defect.

The second-trimester anomaly scan revealed a 2-mm atrial communication without additional abnormalities, prompting referral to an experienced fetal echocardiographer. At 30 weeks’ gestation, detailed fetal echocardiography confirmed the presence of a large foramen ovale, suggestive of an atrial septal defect (Figure 1), and demonstrated cardiomegaly with preserved biventricular systolic function.

**Figure 2:**
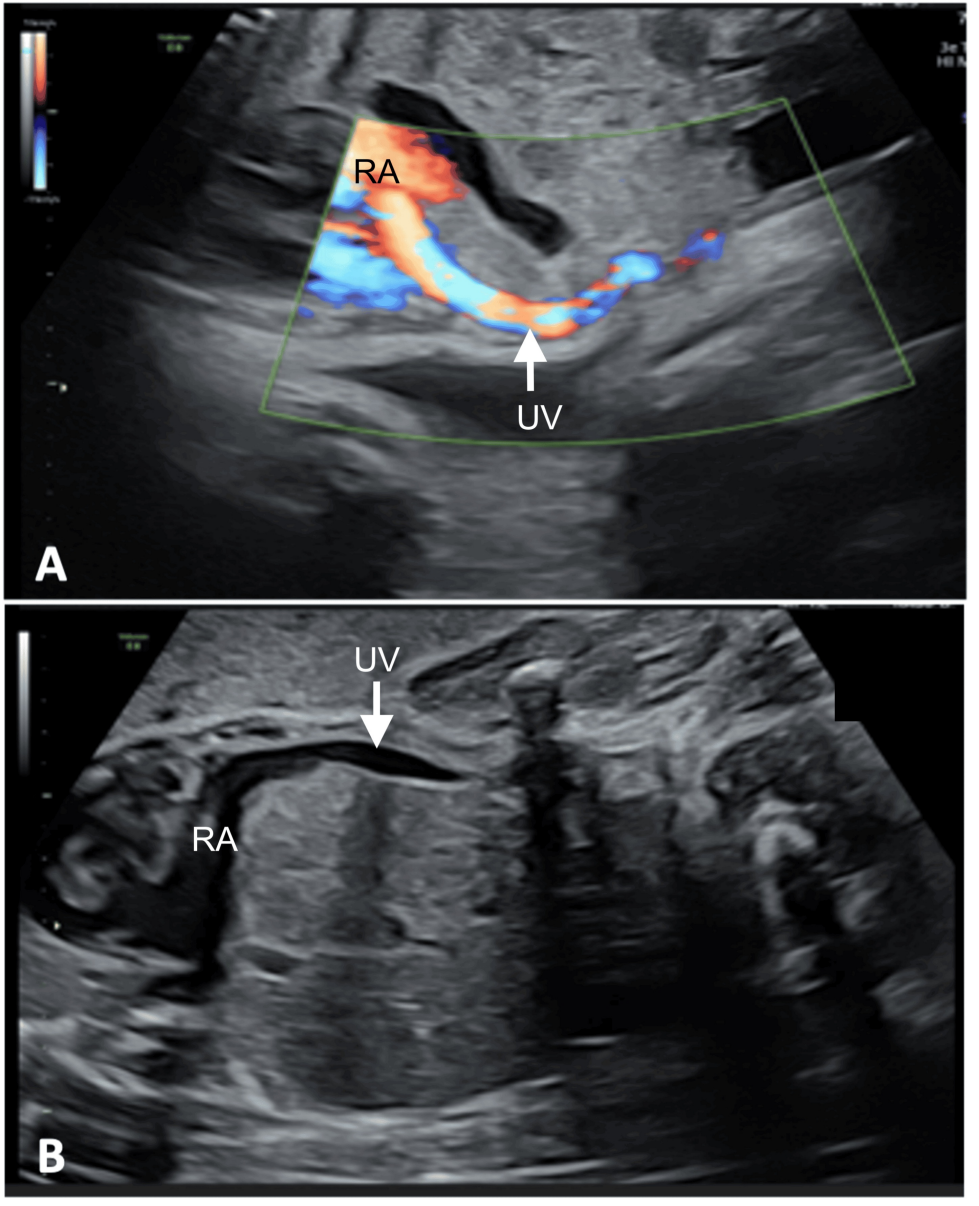
Ultrasound at 30 weeks + 0 days demonstrating a large extrahepatic UV (arrow) draining directly into the RA, consistent with extrahepatic DVA (A) Path of the UV directly into the RA with the turbulent flow between the atria shown by color Doppler. (B) Path of the UV directly into the RA. DVA: ductus venosus agenesis; UV: umbilical vein; RA: right atrium

In the presence of cardiomegaly without an obvious structural explanation, a systematic evaluation of the fetal venous return was undertaken. On sagittal and transverse views, the ductus venosus could not be visualized. Color Doppler imaging failed to demonstrate the characteristic high-velocity aliasing flow between the umbilical vein and the inferior vena cava. Spectral Doppler analysis also did not reveal the typical triphasic waveform of the ductus venosus. Instead, the umbilical vein was observed to course extrahepatically and drain directly into the right atrium (Figure 2). No intrahepatic connection to the portal venous system was identified. These findings confirmed the diagnosis of extrahepatic DVA.

**Figure 3:**
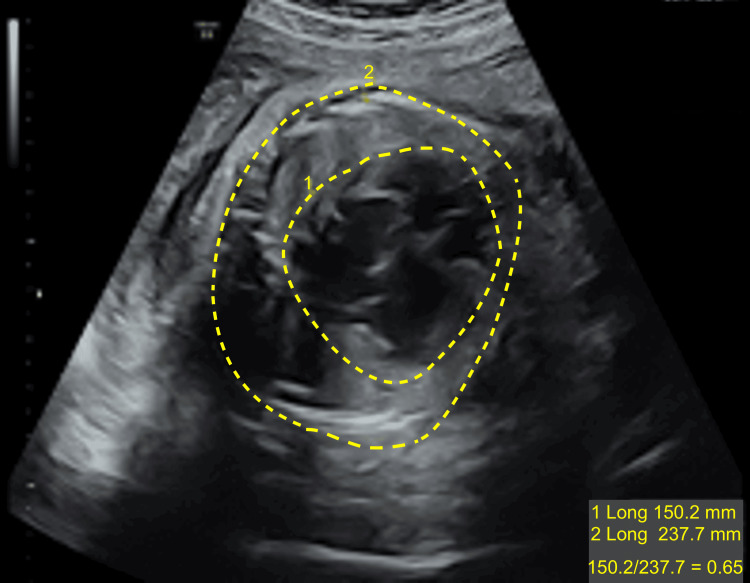
Ultrasound at 30 weeks + 0 days demonstrating cardiomegaly quantified using the cardiothoracic circumference ratio (measured by the point-to-point method of the heart/chest circumference ratio).

Cardiomegaly was quantified using the cardiothoracic circumference ratio measured by the point-to-point method (Figure 3). The ratio was 0.65 ( $\frac{Heart~circumference (mm))}{Chest~circumference (mm))}$ corresponding to $\frac{150.2~mm}{232.7~mm}$ ), exceeding the 95th percentile for gestational age and, thereby, meeting the criteria for cardiomegaly [8,9] (reference ranges and nomograms are available for clinical interpretation [8]). Despite the cardiac enlargement, systolic function remained preserved, and no signs of cardiac decompensation or hydrops were observed at the time of diagnosis.

The case was reviewed at an expert prenatal diagnostic center and by an experienced pediatric cardiologist. Their assessment confirmed our findings and raised suspicion of aortic coarctation based on a reduced aortic annulus and isthmus (aortic annulus z-score −2.60; isthmus z-score −4.08). No additional structural anomalies were identified. Given the known association of DVA with genetic abnormalities, amniocentesis was performed. Molecular karyotyping was normal and revealed a female karyotype.

Prenatal management consisted of weekly ultrasound surveillance, including measurement of the cardiothoracic ratio, assessment of ventricular systolic function, Doppler evaluation of venous return, and systematic screening for signes of hydrops or cardiac decompensation. Fetal growth parameters were also monitored at each visit. Throughout the remainder of the pregnancy, cardiomegaly remained stable, ventricular function was preserved, and no signs of hydrops developed. Fetal growth remained appropriate for gestational age, with biometric parameters consistently between the 40th and 50th percentiles.

Considering the suspected cardiac anomaly, particularly the possible aortic coarctation, and the potential risk of neonatal cardiovascular compromise, delivery was planned at a tertiary care center with specialized pediatric cardiology and surgical support. Labor was induced at 38+2 weeks of gestation. The newborn demonstrated good postnatal adaptation with Apgar scores of 9, 10, and 10 at one, five, and 10 minutes, respectively. Postnatal evaluation excluded aortic coarctation and confirmed a favorable clinical evolution. The chronological sequence of key prenatal examinations, delivery, and postnatal outcome is summarized in Table 1.

**Table 1:** Timeline of key prenatal and postnatal events

Gestational Age	Clinical / imaging event
First trimester ultrasound	Normal, nuchal translucency of 0.8 mm.
Second trimester ultrasound	2-mm atrial communication detected; no other structural anomalies.
30 weeks + 0 days	Specialized ultrasound: extrahepatic ductus venosus agenesis with umbilical vein draining into the right atrium; cardiomegaly (cardiothoracic ratio 0.65, >95th percentile); large foramen ovale suggesting atrial septal defect; preserved systolic function.
30 weeks +2 days	Assessment in a tertiary fetal cardiology center: confirmation of ductus venosus agenesis and suspicion of aortic coarctation (aortic annulus z-score -2.60, isthmus z-score -4.08); no additional cardiovascular malformations.
30–38 weeks	Weekly ultrasound follow-up: measurement of the cardiothoracic ratio, evaluation of ventricular systolic function, Doppler assessment of venous return, systematic screening for signs of hydrops or cardiac decompensation, fetal growth monitoring.
38 weeks + 2 days	Induction of labor in a tertiary care center; vaginal delivery of a female neonate with good postnatal adaptation (Apgar scores 9–10–10).
Postnatal follow-up	No aortic coarctation confirmed postnatally. Favorable subsequent clinical course.

## Discussion

DVA is a rare congenital anomaly of fetal venous development with an estimated prevalence of one in 556 to one in 2532 pregnancies [1,2,10]. Its clinical presentation and prognosis vary depending on the type of agenesis, the presence of associated malformations or genetic abnormalities, and complications arising in utero. The reported case illustrates that the combination of unexplained cardiomegaly and abnormal umbilical venous drainage on color Doppler should prompt systematic evaluation of the ductus venosus and portal venous anatomy. Careful assessment of venous return and cardiac function is essential to establish the diagnosis and guide appropriate prenatal surveillance.

In the fetus of the current case, extrahepatic DVA with direct umbilical venous drainage into the right atrium was associated with cardiomegaly but without signs of cardiac decompensation, hydrops, effusions, or hemodynamic compromise. This case highlights that prognosis depends on multiple factors, including cardiac function, associated anomalies, genetic findings, and potential in utero complications. The stable cardiothoracic ratio and absence of concerning ultrasound features during weekly follow-up supported an expectant management strategy with planned term delivery in a tertiary care center. These findings are consistent with reports indicating that isolated or nearly isolated cases of DVA can have favorable perinatal outcomes [1,2,10-12].

Practical approach to ductus venosus assessment

**Figure 4:**
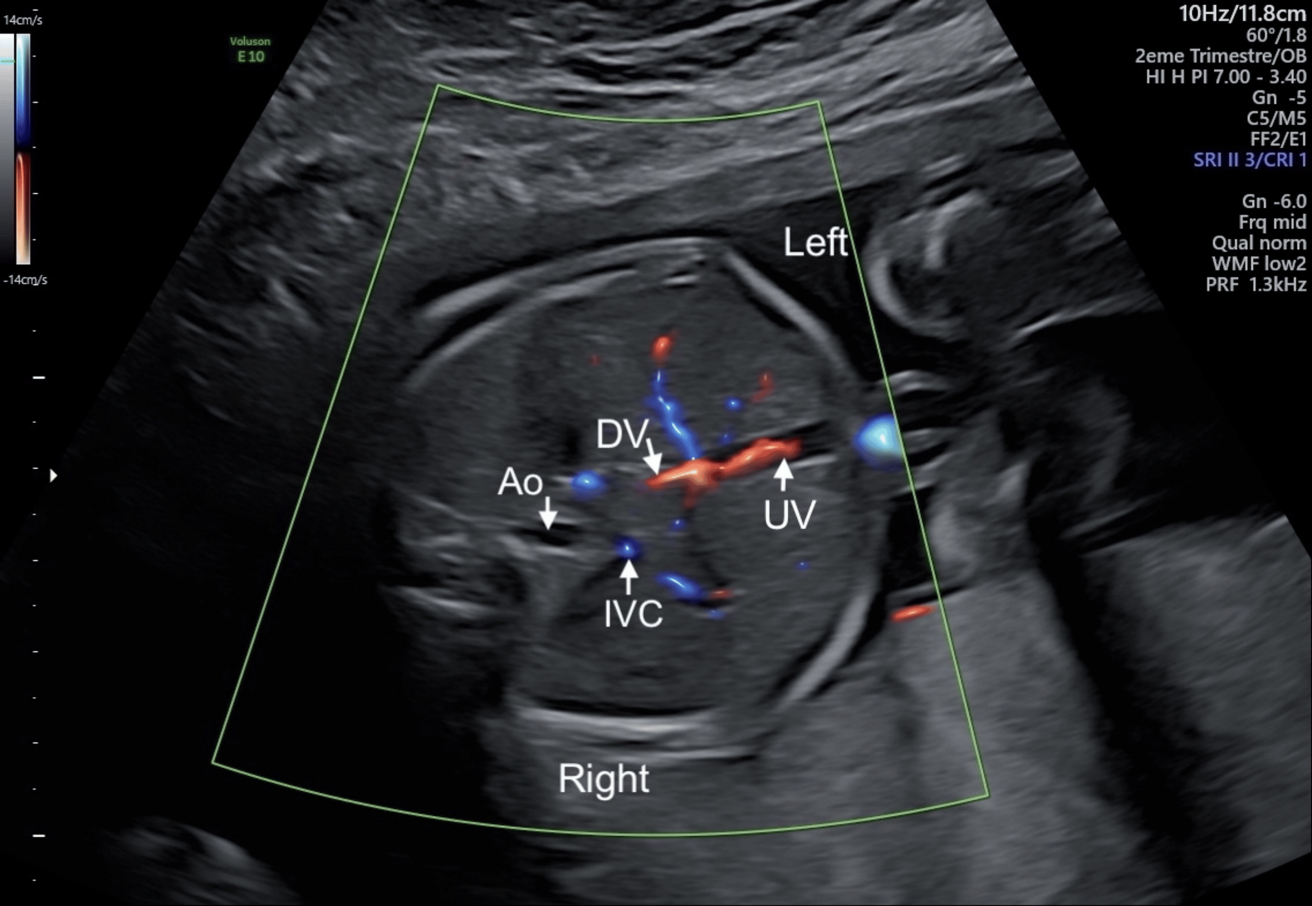
Second-trimester ultrasound (at 22 weeks + 0 day) image showing the ductus venosus to illustrate how to identify a normal ductus venosus from ductus venosus agenesis Mid-sagittal view of the fetal upper abdomen showing the ductus venosus (DV), intrahepatic umbilical vein (UV), inferior vena cava (IVC), and aorta (Ao). Color Doppler highlights the DV coursing toward the IVC. Image Source: Marie Deckx (Author); Image of a patient from the physician's database used with the written consent of the patient

**Figure 5:**
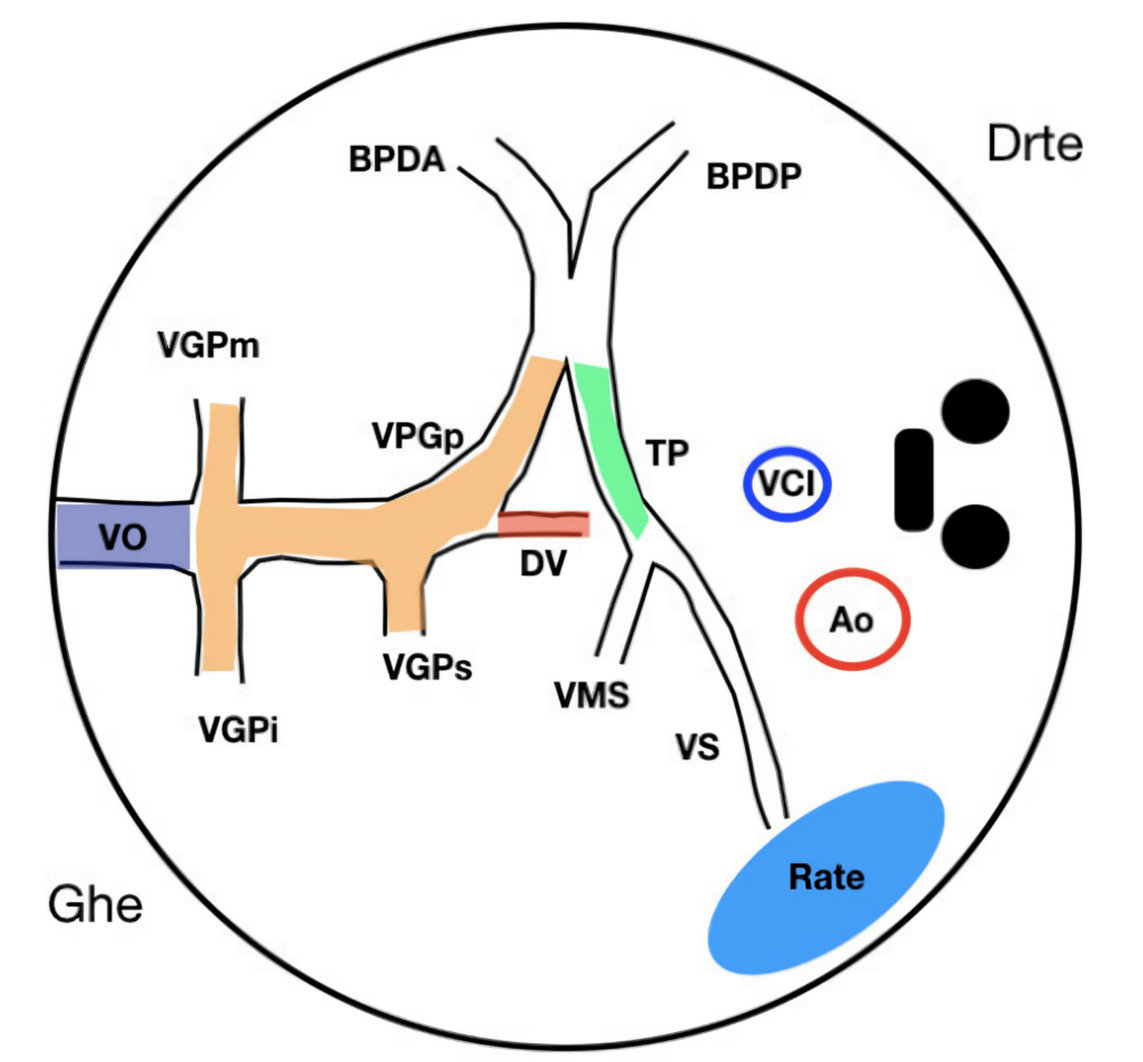
Schematic sagittal view of fetal circulation illustrating the anatomical relationships between the umbilical vein, ductus venosus, portal venous system, and inferior vena cava Ao: aorta; BPDA: right anterior portal vein; BPDP: right posterior portal vein; DV: ductus venosus; Drte: right; Ghe: left; Rate: spleen; TP: portal vein; VCI: inferior vena cava; VGPi: inferior branch of left portal vein; VGPm: medial branch of left portal vein; VGPs: splenic portal branch; VPGp: left portal vein; VO: umbilical vein; VMS: superior mesenteric vein; VS: splenic vein. Figure Source: Reprinted from Jacquier et al. [13], Copyright (2026), with permission from Elsevier. Expansions of the abbreviations are presented in English; labels within the figure remain in the original language (French).

In routine practice, the ductus venosus is visualized in a right ventral mid-sagittal view of the fetal trunk, using maximum magnification so that the fetal thorax and upper abdomen occupy most of the image. Color Doppler is applied to identify the intrahepatic umbilical vein and the ductus venosus as it courses cranially toward the inferior vena cava. The corresponding ultrasound image and schematic representation are shown in Figures 4, 5. Pulsed Doppler recording uses the smallest possible sample volume (0.5-1.0 mm) positioned in the upper part of the ductus venosus, with the insonation angle kept as close to zero as possible (<30° ideally) to avoid contamination from adjacent vessels. The waveform should be obtained during periods of fetal quiescence and shows forward flow during ventricular systole (S‑wave), diastole (D‑wave), and atrial contraction (a‑wave) [6].

Anatomical variants and timing of diagnosis

**Figure 6:**
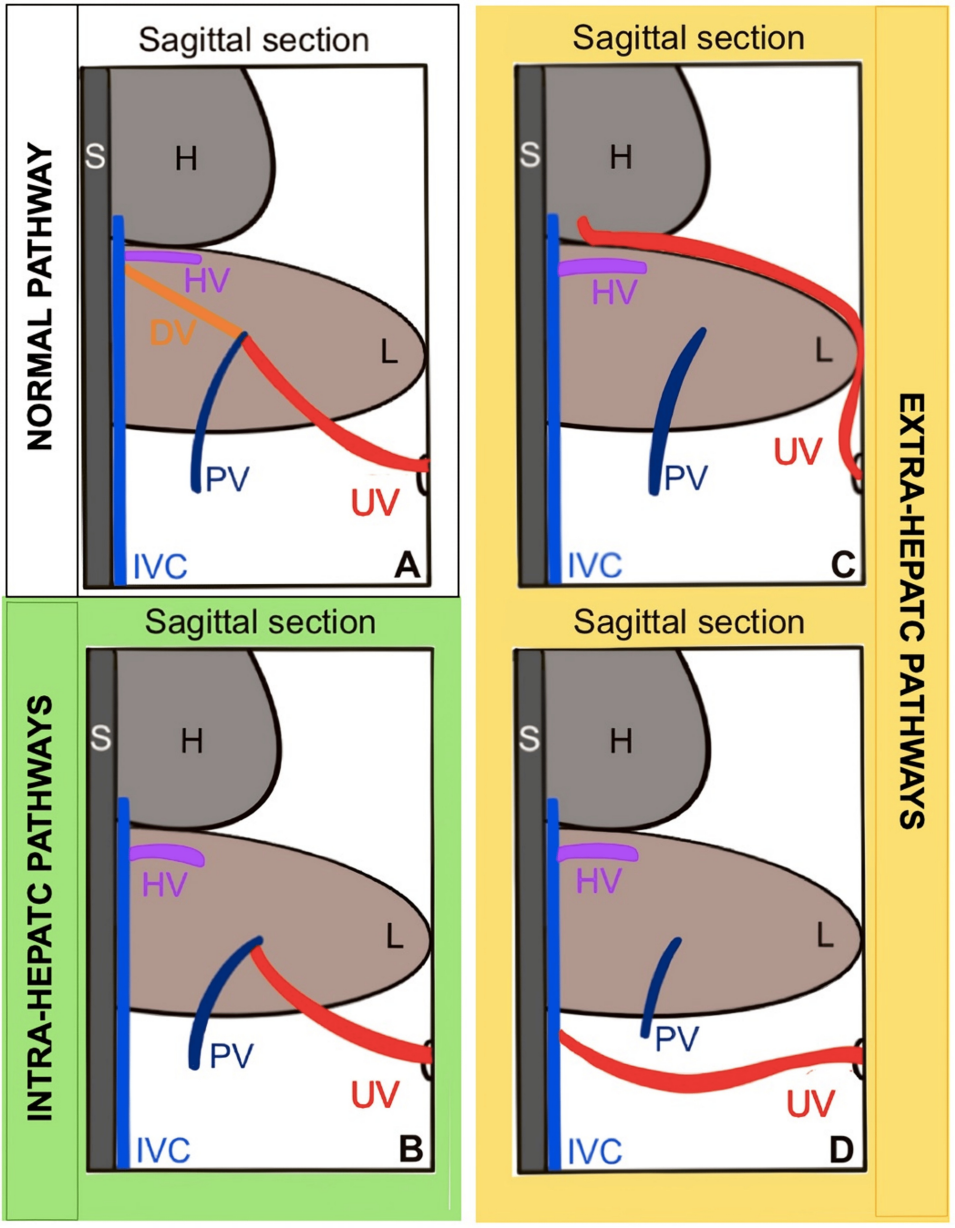
Schematic representation of fetal hepatic, portal and systemic circulation, illustrating the most common forms of DVA (A) Normal anatomy; (B) DVA – intra-hepatic pathway : umbilical vein drains into the portal vein; (C) DVA – extra-hepatic pathway : umbilical vein drains into the right atrium; (D) DVA – extra-hepatic pathway : umbilical vein drains into the inferior vena cava DV: ductus venosus; H: heart; HV: hepatic veins; IVC: inferior vena cava; L: liver; PV: portal vein; S: spine; UV: umbilical vein; DVA: ductus venosus agenesis Image credit: Author; created with Procreate app on iPad (Apple Inc., Cupertino, California, United States).

Diagnosis of DVA is most commonly made during the second trimester [1, 2,10,12] owing to the increasingly routine evaluation of the fetal venous system and widespread use of color Doppler during the morphology scan [1,2]. This timing also coincides with the frequent detection of associated anomalies at this stage [1,2,12]. Several classifications of DVA have been proposed. The most widely used in clinical practice is a dichotomous system distinguishing intrahepatic forms (via the portal venous system) from extrahepatic forms (with complete bypass of the hepatic circulation). This classification was first described by Gembruch et al. [14] and later refined by Nagy et al. [4], who emphasized its prognostic significance. Overall, the literature suggests that extrahepatic forms are more common than intrahepatic forms [1,2,10]; for example, Pacheco et al. reported 60.8% extrahepatic versus 39.2% intrahepatic [1]. However, these figures should be interpreted with caution, as extrahepatic pathways are generally easier to identify on prenatal ultrasound, potentially leading to underdiagnosis of intrahepatic variants [2]. In extrahepatic cases, the umbilical venous return most frequently drains into the right atrium (with Pacheco et al. reporting this in 43.6% of cases [1]) or into the inferior vena cava (with Pacheco et al. reporting this in 34.0% of cases [1]). Less commonly, it may connect to the iliac vein (common, right, or left), coronary sinus (typically dilated), renal vein, left atrium, or superior vena cava. In intrahepatic variants, drainage occurs most often into the portal vein (with Pacheco et al. reporting this in 28.9% of cases), and more rarely into the portal sinus or a hepatic vein [1]. Figure 6 illustrates the most frequently encountered anatomical variants of ADV.

Associated malformations, genetic anomalies, and in utero complications in the literature

Available evidence indicates that more than half of the fetuses with DVA present at least one additional malformation, with reported rates of associated anomalies exceeding 70% in several cohorts (72.9% (210/288) in Piemonti et al. [2], 75.8% (25/33) in Mash et al. [12], and 82.9% (340/410) in Pacheco et al. [1]). However, this trend is not uniform, as other series reported higher rates of isolated DVA; the systematic review by Moaddab et al. found isolated cases in 56.8% (147/259) [10]. These discrepancies likely reflect differences in inclusion criteria, referral patterns to tertiary centers, and the systematic use of detailed fetal echocardiography and the depth of genetic investigation, as comprehensive testing often reveals associated genetic conditions in cases initially classified as isolated.

Cardiac anomalies are the most frequently associated malformations, reported in 35.9-55.2% of cases, depending on the series [1,2,12]. In our case, a suspicion of aortic coarctation was raised in utero but excluded at birth; this anomaly is reported in approximately 3.5% of DVA cases [10]. Extracardiac malformations have also been described, including musculoskeletal anomalies (facial, limb, or vertebral abnormalities), gastrointestinal defects (tracheoesophageal fistula, tracheal or esophageal atresia, anal atresia), genitourinary anomalies (renal agenesis, hydronephrosis, micropenis, absent bladder), and central nervous system abnormalities [1,2,10]. Their distribution is summarized in Table 2. Cardiac anomalies are isolated in 23-25.5% of cases, extracardiac anomalies in 7.3-26.5%, and combined anomalies in 10.4-27.5% [1,10]. Importantly, no significant difference has been demonstrated between the type of malformation and the type of DVA (intrahepatic vs. extrahepatic) [15].

**Table 2:** Categories of extracardiac malformations associated with DVA according to three published studies Percentages are calculated based on the total number of cases reported in each study. Categories may overlap when multiple anomalies were present in the same fetus. DVA: ductus venosus agenesis

Extracardiac malformations	Pacheco et al. [1] (n=340), n (%)	Piemonti et al. [2] (n=288), n (%)	Moaddab et al. [10] (n=258), n (%)
Musculoskeletal malformations	70 (20.6%)	30 (10.4%)	23 (8.9%)
Gastrointestinal malformations	31 (9.1%)	28 (9.7%)	20 (7.7%)
Genitourinary malformations	31 (9.1%)	23 (8.0%)	16 (6.2%)
Central nervous system malformations	16 (4.7%)	24 (8.3%)	8 (3.1%)

Overall, the interpretation of the literature is limited by methodological heterogeneity. Most available data are derived from small cohorts, extended recruitment periods, tertiary centers, or compilations of published cases, which introduce selection bias and may overestimate complex cases, though these specialized cohorts offer the advantage of better characterizing severe phenotypes. In addition, advances in ultrasound technology and expertise have also improved detection, likely leading to underdiagnosis of isolated or less severe forms in earlier series. Moreover, a non-negligible proportion of cases result in termination of pregnancy (22% (57/259) in Moaddab et al. [10], 24.1% (68/282) in Piemonti et al. [1]), which excludes affected fetuses from final survival statistics and further biases prevalence estimates.

Genetic anomalies are reported in 10.2-34% of DVA cases [1,2,11,15]. The most frequent are summarized in Table 3, and include trisomy 21, monosomy X (Turner syndrome), and trisomy 18 [1,2,10,15]. Other syndromes have also been described, most consistently Noonan syndrome, with an estimated prevalence of 0.9-2.4% [1,2,10,12]. When a genetic anomaly is present, structural malformations frequently co-occur, with up to 84% of cases exhibiting at least one additional malformation [2]. This supports the recommendation for systematic genetic testing in all cases of DVA, even when apparently isolated. At a minimum, testing should include conventional karyotyping [2,12] ideally complemented by chromosomal microarray, targeted sequencing (e.g., RASopathy gene panel for Noonan syndrome), or exome sequencing [12]. However, these data should be interpreted with caution due to significant methodological heterogeneity. Many reported cases did not undergo genetic testing, reflecting the gradual evolution of diagnostic techniques over recent years. Among those tested, methods ranged from conventional karyotyping to more advanced approaches, such as microarray or exome sequencing. Consequently, these methodological differences limit the reliability of reported frequencies and likely result in under- or overestimation of the true prevalence of genetic anomalies in DVA.

**Table 3:** Reported genetic anomalies associated with DVA in four published studies Percentages represent the proportion of genetically tested cases in each cohort. Not all fetuses underwent comprehensive genetic testing, and testing modalities varied between studies (karyotyping, microarray, exome sequencing), which may influence reported prevalence rates. “Other” refers to chromosomal deletions, duplications, translocations, and rare genetic syndromes as specified in the original publications.

Genetic anomalies	Pacheco et al. [1] (n=141), n (%)	Piemonti et al. [2] (n=165), n (%)	Moaddab et al. [10] (n=154), n (%)	Strizek et al. [15] (n=119), n (%)
Trisomy 21	11 (7.8%)	16 (9.7%)	10 (6.5%)	10 (8.4%)
Monosomy X (Turner syndrome)	12 (8.5%)	6 (3.6%)	11 (7.1%)	5 (4.2%)
Trisomy 18	6 (4.3%)	6 (3.6%)	6 (3.9%)	2 (1.7%)
Other, including chromosomal deletions	19 (13.5%)	29 (17.6%)	11 (7.1%)	7 (5.9%)
Total	48 (34.0%)	57 (34.5%)	38 (24.7%)	24 (10.2%)

Beyond structural malformations, DVA exposes the fetus to several potential in utero complications, most commonly cardiomegaly (24.1% (82/340) in Pacheco et al. [1], 24.7% (64/259) in Moaddab et al. [10]), cardiac decompensation, amniotic fluid abnormalities (polyhydramnios and oligohydramnios), and hydrops fetalis [1,2,10]. Fluid effusions may also be confined to a single compartment, such as the pleural cavity, pericardium, or subcutaneous tissue [1,10]. These manifestations may lead to the initial diagnosis of DVA [8] and often worsen in the third trimester [1], highlighting the importance of close ultrasound monitoring. In addition, certain ultrasound findings, such as cardiomegaly, single umbilical artery, or increased nuchal translucency, are associated with a higher risk of chromosomal abnormalities and venous malformations, warranting systematic evaluation of the ductus venosus in such cases [10]. Furthermore, cardiomegaly is more frequently observed in extrahepatic forms of DVA [15].

Perinatal prognostic factors and perinatal outcomes

Perinatal prognosis largely depends on the presence of associated morphological anomalies, specific ultrasound findings (e.g., hydrops), genetic status (notably abnormal karyotypes, which worsen prognosis), prematurity [2,10], and type of drainage, with extrahepatic forms of DVA being associated with poorer outcomes [1]. In contrast, isolated DVA generally carries a favorable prognosis [1,2,10,12,15] with lower rates of genetic anomalies [12], although perinatal mortality can reach 6% in some series [2]. Postnatal complications have also been described, including congestive heart failure, pulmonary edema, focal nodular hyperplasia, and hepatic tumors [2,10]. These findings underscore the need for a comprehensive evaluation of associated malformations and genetic anomalies to guide obstetric follow-up, parental decision-making, and neonatal management.

Prenatal counselling and multidisciplinary perinatal management

Prenatal counselling for DVA should provide a comprehensive overview of its rarity, heterogeneous prognosis, and the key factors outlined influencing outcomes: venous drainage pattern, associated structural and genetic anomalies, and potential in utero complications [16]. Parents must be informed that while isolated cases generally carry a favorable prognosis under close monitoring, extrahepatic forms, chromosomal abnormalities, and evolving hydrops are associated with higher risks of adverse perinatal outcomes, including intrauterine fetal death (approximately 7%) [2]. The possibility of termination of pregnancy should also be discussed objectively alongside expectant management options.

Management requires a multidisciplinary approach involving obstetrics specialists, fetal/pediatric cardiologists, clinical geneticists, and neonatologists. Referral to a tertiary care center with expertise in fetal echocardiography, advanced genetic testing, and neonatal cardiology services is essential [1,2,10]. Systematic genetic testing is strongly recommended even in apparently isolated cases and should include, at minimum, conventional karyotyping [2,12], ideally complemented by chromosomal microarray, targeted sequencing, or exome sequencing [12].

Close monitoring is required due to the non-negligible risk of rapid fetal cardiac deterioration [2]. In the absence of standardized guidelines, we recommend weekly ultrasound surveillance focusing on cardiothoracic circumference ratio, cardiac function, amniotic fluid volume, fetal growth, and early signs of hydrops or cardiac decompensation [10]. It is also important to systematically evaluate the ductus venosus in cases of fetal cardiomegaly [2,10], as cardiomegaly may be one of the earliest sonographic signs of DVA [1].

Delivery timing and mode should be individualized based on ultrasound findings and clinical stability. Piemonti et al. reported a mean gestational age at delivery of 36 weeks [2]. Vaginal delivery was most common; among vaginal births, 83.8% (31/37) occurred at term (≥37 weeks) and 16.2% (6/37) preterm. Among cesarean sections, only 41.2% (14/34) were at term versus 58.8% (20/34) preterm [2]. In the absence of concerning features, such as hydrops, cardiac decompensation, or growth restriction, as in the present case, term vaginal delivery in a tertiary care center with neonatal cardiology support seems appropriate. Conversely, evidence of progressive cardiac dysfunction or other worrisome signs should prompt earlier delivery planning.

Methodological considerations

The limitations of the study, including tertiary referral bias, heterogeneous genetic testing protocols, extended recruitment periods, and high rates of pregnancy termination, affect the reliability of reported prevalence estimates for malformations and genetic disorders. These methodological differences prevent direct extrapolation to contemporary practice, where advances in ultrasound detection and comprehensive genomic testing may influence observed frequencies. Consequently, reported outcomes should guide, but not dictate, prenatal counselling, with management decisions individualized based on case-specific findings, serial monitoring, and multidisciplinary expertise. Prospective multicenter studies using standardized diagnostic criteria, genetic protocols, and complete postnatal follow-up are needed to improve the accuracy of prognostic counselling.

## Conclusions

DVA is a rare anomaly diagnosed by ultrasound and color Doppler, most often during the second trimester. Its reported prevalence is expected to increase with advances in imaging techniques and systematic evaluation of the venous system. Prognosis varies widely and depends on the drainage pattern (with extrahepatic forms generally associated with poorer outcomes), associated structural malformations (particularly cardiac anomalies), associated genetic abnormalities, in utero complications (such as cardiomegaly, cardiac decompensation, or hydrops), and prematurity. These factors underscore the importance of comprehensive fetal morphological and genetic assessment, even in apparently isolated cases.

Isolated DVA generally carries a favorable prognosis; however, close weekly ultrasound surveillance remains standard due to the risk of rapid deterioration. In the absence of standardized guidelines, individualized multidisciplinary care is essential, integrating close ultrasound follow-up, appropriate genetic testing, detailed parental counselling regarding prognostic variability and termination options, and delivery planning at a tertiary center. Term vaginal delivery may be appropriate for stable cases, whereas earlier intervention is warranted in fetuses showing signs of decompensation. There is a need for prospective multicenter studies with standardized diagnostic criteria and follow-up to refine prenatal counselling and optimize management protocols.
